# Neuronal correlates of personal space intrusion in violent offenders

**DOI:** 10.1007/s11682-016-9526-5

**Published:** 2016-03-02

**Authors:** Anne Schienle, Albert Wabnegger, Mario Leitner, Verena Leutgeb

**Affiliations:** 1 Clinical Psychology, BioTechMedGraz, University of Graz, Universitätsplatz 2/DG, 8010 Graz, Austria; 2 Graz-Karlau State Correctional Facility, Herrgottwiesgasse 50, 8200 Graz, Austria

**Keywords:** Violent offenders, Personal space intrusion, Insula, fMRI

## Abstract

Personal space (PS) is defined as the imagery region immediately surrounding our body, which acts as safety zone. It has been suggested that PS is enlarged in violent offenders and that this group shows an enhanced sensitivity to the reduction of interpersonal distance. In the present fMRI study high-risk violent offenders and noncriminal controls were presented with photos of neutral facial expressions by men and women. All images were shown twice, as static photos, and animated (i.e., appearing to approach the subject) in order to simulate PS intrusion. Approaching faces generally provoked activation of a fronto-parietal network and the insula. Offenders responded with greater insula activation to approaching faces, especially when the person was male. Insular activation has been recognized before as a neuronal correlate of potential threat and harm detection in PS. The increased reactivity of violent offenders is possibly a result of their hostile attribution bias.

**Electronic supplementary material:**

The online version of this article (doi:10.1007/s11682-016-9526-5) contains supplementary material, which is available to authorized users.

## Introduction

Personal space (PS) is defined as the imaginary region immediately surrounding our bodies, which we regard as psychologically ours. This region functions as a safety zone into which others may not intrude without causing discomfort (Holmes and Spence 2004).

One widely used method to investigate PS intrusion consists of the presentation of stimuli that are expanded (i.e., appearing to approach the subject). During a typical experiment the participants view for example non-animated photos of human faces with neutral expressions, which are then increased in apparent size (‘zoomed-in’) in the intrusion condition (e.g., Holt et al. 2014). This design has been applied in functional magnetic resonance imaging (fMRI) studies with healthy individuals (Holt et al. 2014), patients with borderline personality disorder (Schienle et al. 2015), and a patient with complete amygdala atrophy (Kennedy et al. 2009). The studies showed that a fronto-parietal network responds to changes in interpersonal distance. Several parietal regions (inferior/superior parietal cortex, primary/secondary somatosensory cortex (SI, SII)) and the premotor cortex demonstrated greater responses to approaching relative to static or withdrawing faces (Holt et al. 2014; Schienle et al. 2015). These findings are in line with previous studies on the neuronal representation of near space in parietal and frontal areas (for review, see Brozzoli et al. 2014). In addition, PS intrusion elicited amygdala activation in healthy individuals (Kennedy et al. 2009; Schienle et al. 2015). In contrast, a patient with a complete amygdala lesion had lost any sense of PS. Even at the point of touching the experimenter, the patient reported no discomfort (Kennedy et al. 2009).

Other studies on PS processing focused on the emotional context of intrusion (e.g., Lloyd et al. 2006; Schienle et al. 2015). It could be shown that the approach of an aversive or painful stimulus (e.g., a knife, a face with a disgusted or angry expression) within the space surrounding the body not only recruited visuo-tactile networks, but also limbic regions (e.g., anterior cingulate cortex, insula, amygdala). The latter activation is related to the evaluation of the motivational relevance of the stimulation in order to initiate appropriate defensive actions that help to avoid or minimise harm (Lloyd et al. 2006). These findings underline that the size of PS shows intra-individual variability associated with the affective value of the approaching stimulus.

Moreover, PS shows inter-individual variability. For example, individuals characterized by antisocial and aggressive behavior have an increased PS. They prefer a greater interpersonal distance during social interactions (e.g., McGurk et al. 1981; Curran et al. 1978; Gilmour and Walkey 1981; Lawrence and Andrews 2004). This is particularly true for offenders who committed personal crimes that resulted in physical harm to another person compared to those convicted with property crimes (e.g., Booream et al. 1977).

The enhanced sensitivity of violent offenders to reductions of interpersonal distance might be associated with their hostile attribution bias. This bias consists of the tendency to perceive malicious intentions in others. It is a skewed system of appraisal and expectancies that can predict aggressive behaviour especially in ambiguous situations (Epps and Kendall 1995).

Despite the well-established importance of PS in individuals characterized by aggressive/antisocial behaviour, the underlying neuronal correlates are completely unknown. Therefore, we simulated PS intrusion by means of approaching neutral (ambiguous) facial expressions of men and women. The neuronal and affective responses to the animated images were contrasted with non-animated facial expressions and compared between high-risk violent offenders and controls. We predicted that the offenders would experience more arousal and negative valence when confronted with PS reduction (approaching faces) and would show stronger activation of the amygdala, insula and a fronto-parietal network (premotor cortex, superior/inferior parietal cortex, SI/SII) than controls.

## Method

### Sample

We investigated 17 male violent offenders (inmates) from a maximum security prison located in Graz (Austria) and 18 male, non-delinquent controls with comparable mean age (offenders: M = 34.82 years (SD = 12.54), controls: M = 37.89 years (SD = 9.21); t(33) = 0.83, *p* = .41) and educational status (years of education; offenders: M = 11.18 years (SD = 2.07), controls: M = 11.78 (SD = 1.73; t(33) = 0.93, *p* = .39). Violent offences included death of another person or serious bodily harm. Sexual offenses led to exclusion from the sample. The inmates had spent on average 61.3 months in prison (range: 19–183 months) and were to be released to the community in the near future (conditional release or completion of sentence). A comprehensive clinical interview (according to Wittchen et al. (1997)) with additional risk assessment of violent recidivism had been performed with each offender by experienced forensic psychologists or psychiatrists. Clinically relevant current symptoms of depression, bipolar disorder, psychosis, attention deficit hyperactivity disorder, anxiety disorders (e.g., social anxiety disorder), personality disorders (with the exception of antisocial personality disorder; *n* = 5) as well as somatic conditions (e.g., hyper- or hypothyroidism, history of severe head injury) led to exclusion from the sample. Moreover, offenders with a history of substance and/or alcohol abuse during imprisonment were excluded.

Community healthy control participants were recruited via announcements in a local newspaper. Control group participants who had been convicted for any crime, or had a history of a mental/somatic disorder or substance/alcohol abuse, were excluded.

All participants provided written informed consent after receiving a full explanation of the test procedure. The study had been approved by the ethics committee of the University of Graz.

### Pictures and design

The participants were presented with a total of 50 pictures from the Karolinska Directed Emotional Faces (Lundqvist et al. 1998) which showed neutral facial expressions of 25 men and 25 women. In an event-related approach each image was presented for 3000 ms with a mean inter-trial-interval of 5000 ms (range: 4000–6000 ms); half of the pictures were presented as ‘static’ photos; the others as ‘approaching’. In the latter condition the original picture was enlarged (factor: 2.75) up to point that only the region involving the mouth and the eyes could be seen. This gave the impression that the approaching person almost touched oneself. The stimulus sequence was random. At the end of the task 8 randomly chosen pictures (4 men, 4 women) were presented again and rated according to elicited arousal and valence on 9-point-scales (9 = very arousing, negative).

### Procedure

The study was conducted at the University of Graz. The offenders were either given permission to leave prison on a day-release, or were escorted to the University by officials of the Graz-Karlau State Correctional Facility, Graz, Austria.

All participants answered the Hare Psychopathy Checklist-Revised (PCL-R; Hare 2003) to quantify the degree of psychopathic traits. The PCL-R consists of 20 items, which are rated on a 3-point-scale by means of a semi-structured interview. There are two factors: Factor 1 is labelled ‘selfish, callous and remorseless use of others’, whereas Factor 2 is labeled ‘chronically unstable, antisocial and socially deviant lifestyle’. Individuals who score high on this factor show proneness to boredom, poor behavioral control, and a long history of delinquency. The cut-off is 30 for the PCL-R sum score. Inter-rater reliabilities (Pearson’s correlations) for the sum score and subscales (Factor 1 and 2) of the PCL-R were sufficiently high and ranged for offenders from *r* = .80 to .88, and for controls from .81 to .96.

Moreover, all participants were shown two black silhouettes of a man representing the participant from the side (height = 50 mm) on a sheet of paper. They were asked to draw a bubble around the silhouette representing the distance they would like to keep from a male and a female stranger of about their age (e.g., a shop assistant), respectively. The bubble diameter (mm) was used as an indicator of PS size. A pilot study had indicated retest reliabilities for this measure (2-week interval) ranging between .79–.84.

### fMRI recording

The fMRI session was conducted with a 3 T scanner (Skyra, Siemens, Erlangen, Germany). Functional runs were acquired using an echo-planar imaging protocol (number of slices: 35, descending, flip angle =90°, slice thickness: 3 mm; slice spacing: 3.99 mm; matrix: 64 × 64; TE = 30 ms; TR = 2290 ms; FoV: 192; in-plane resolution =3^x^3^x^3 mm). All analyses were conducted using SPM 12 (Wellcome Department of Cognitive Neurology, London). Three volumes from the beginning of the time series were discarded to account for saturation effects.

First, the functional data were motion-corrected via realignment and acquisition timing was accounted in the slice timing step. Individuals T1 images were coregistered to their functional data. Afterwards coregistered T1 images got segmented into grey matter (GM), white matter (WM) and cerebrospinal fluid. To increase the accuracy of inter-subject alignment we applied the ‘Fast Diffeomorphic Registration Algorithm’ (DARTEL) and used the existing IXI-template implemented in the VBM8 toolbox. Functional images were then normalized to MNI-space (3 mm isotropic voxel), and smoothed with an 8 mm isotropic Gaussian kernel. We compiled vectors for each event of interest (picture onset) and entered them into the design matrix to model event-related responses by the canonical hemodynamic response function in the first level stage. Data were high pass filtered (128 s). Temporal sphericity was controlled by an AR(1) process with consecutive prewhitening of the data.

### Statistical analyses

The ratings for the pictures (valence, arousal) were analyzed with repeated measures ANOVAs (SPSS; version 22) with the within-subject factors ‘Poser gender’ (Male, Female) and ‘Motion’ (Static, Approaching), and the between-subject factor ‘Group’ (Offenders, Controls). For the PS measure, we conducted an ANOVA with the factors ‘Poser gender’ and ‘Group’. Significant effects were followed up with post-hoc t-tests (with Bonferroni correction) and effect sizes η2p were computed. PCL scores were compared between the two groups via t-tests.

For the fMRI data we computed an analysis of variance with GLM-Flex (http://mrtools.mgh.harvard.edu/index.php/Main_Page) with the factors ‘Motion’ (Static, Approaching), Poser gender (Male, Female), and ‘Group’ (Offenders, Controls). Statistically significant main effects and interaction effects were followed up by one-dimensional t-contrasts. We conducted exploratory whole-brain voxel intensity tests as well as region of interest (ROI) analyses for the amygdala, the insula, the premotor cortex, and parietal regions (inferior parietal region, primary/secondary somatosensory cortex (SI, SII)). These regions had been selected based on previous findings on personal space processing (e.g. Kennedy et al. 2009; Schienle et al. 2015; Holt et al. 2014; Lloyd et al. 2006). In addition, there is mounting evidence that antisocial individuals show functional abnormalities in prefrontal cortex regions (Yang and Raine 2009). Therefore the orbitofrontal cortex (OFC) and dorsolateral/ventrolateral prefrontal cortex (DLPFC, VLPFC) were considered further ROIs.

To extract peak values from clusters we used the peak_nii script by Donald McLaren and Aaron Schultz (https://www.nitrc.org/projects/peak_nii). In addition to the analyses of variance, we conducted multiple regressions analysis in SPM 12 separately for each group to correlate PCL-R scores (sum score; Factor 1, Factor 2) with activation in those ROIs differentiating both groups. For the present study we used ROIs from the automated anatomical labeling (AAL) template and from the Juelich histological atlas (Eickhoff et al. 2005). The ROIs derived from the AAL-template were constructed with the WFU PickAtlas (version 2.4; Wake Forest University School of Medicine). For the ROI analyses we applied a height threshold of *p* < .005 (uncorrected) and an extent threshold of 5 voxels. Results reported are based on family-wise error (FWE) correction for voxel intensity tests (p_FWE_ < .05; small volume correction).

## Results

### Self-report

#### PCL-R

Offenders (M = 17.8, SD = 8.3) and controls (M = 1.67, SD = 1.54) differed in their PCL-R sum scores t(33) = 8.01, *p* < .001). The scores ranged between 0 and 29 across all subjects. Also, scores (M, SD) for Factor 1 (Offenders: 5.5 ± 3.4; Controls: 0.6 ± 0.7) and Factor 2 (Offenders: 9.7 ± 6.1; Controls: 1.1 ± 1.4) showed significant group differences (both p’s < .001).

#### PS size

Only the main effect for Poser gender was statistically significant (F(1,33) = 8.17, *p* = .007, η2p = .20; all other effects *p* > .30). The preferred distance to a woman was smaller compared to a man (*p* < .01).

#### Affective ratings

The analysis of variance for valence revealed a significant main effect for Poser gender (F(1,33) = 16.39, *p* < .001, η2p = .34) and a significant interaction Poser gender x Motion (F(1,33) = 6.15, *p* = .019, η2p = .16). Post-hoc t-tests showed that in the approaching condition male faces received more negative valence ratings than female faces (t(33) = −4.41, *p* < .001), which was not the case in the static condition (t(33) = −1.45, *p* = .157). Generally, female faces were perceived as less negative than male faces (*p* < .01).

For arousal, we observed a significant main effect for Motion (F(1,33) = 4.40, *p* = .041, η2p = .12). Approaching faces were judged as more arousing than static ones (*p* < .01). There were no group differences in affective ratings (see supplementary Table S1).

### fMRI data

The analysis of variance for the selected ROIs revealed significant main effects for Group, Motion and Poser gender (see Table 1). The post-hoc t-contrasts showed greater inferior parietal activation in controls relative to offenders (effect: Group), and greater activation for the contrast Approaching > Static in fronto-parietal regions (premotor cortex, SI, DLPFC, superior/inferior parietal region) as well as in the insula (effect: Motion). Female faces elicited greater OFC activation than male faces (effect: Poser gender).

**Table 1:** Results of the analysis of variance for regions of interests

	H	x	y	z	F	Post-hoc t tests	p(FWE)	Cluster size
Main effect GROUP								
Controls > Offenders								
Inferior parietal region	R	45	−36	24	10.90	3.30	0.015	16
Main effect POSER GENDER								
Female > Male								
OFC	R	42	24	−9	15.43	3.93	.0110	255
Main effect MOTION								
Approaching > Static								
DLPFC	R	36	−2	51	32.74	5.72	0.001	445
Premotor cortex	R	51	6	45	23.25	4.82	0.006	40
SI	L	−32	−42	55	35.77	5.98	< 0.001	39
SI	R	32	−43	53	20.66	4.55	0.003	110
Insula	L	−33	15	9	23.71	4.87	0.003	315
Superior parietal region	R	15	−54	66	38.26	3.61	0.018	30
Superior parietal region	L	−3	45	57	13.05	3.61	0.006	9
Inferior parietal region	L	−60	−30	24	16.70	4.09	0.002	50
Interaction: POSER GENDER X MOTION								
Male > Female: Approaching > Static								
Amygdala	L	−15	−6	−18	9.88	3.14	0.024	12
Inferior parietal region	L	−51	−54	48	11.43	3.38	0.017	43
Interaction: GROUP x MOTION								
Offenders > Controls: Approaching > Static								
Insula	L	−33	21	9	19.56	4.42	0.009	323
Interaction: GROUP x POSER GENDER X MOTION								
Offender > Controls: Male > Female: Approaching > Static								
Insula	R	39	−12	15	17.10	4.18	0.017	267

H Hemisphere, x,y,z MNI coordinates, F-values of analyses of variance, post-hoc t-tests with p (corrected for family-wise error (FWE)); cluster size: number of voxels in associated cluster; SI primary somatosensory cortex; OFC Orbitofrontal cortex; DLPFC Dorsolateral prefontal cortex

**Fig. 1:**
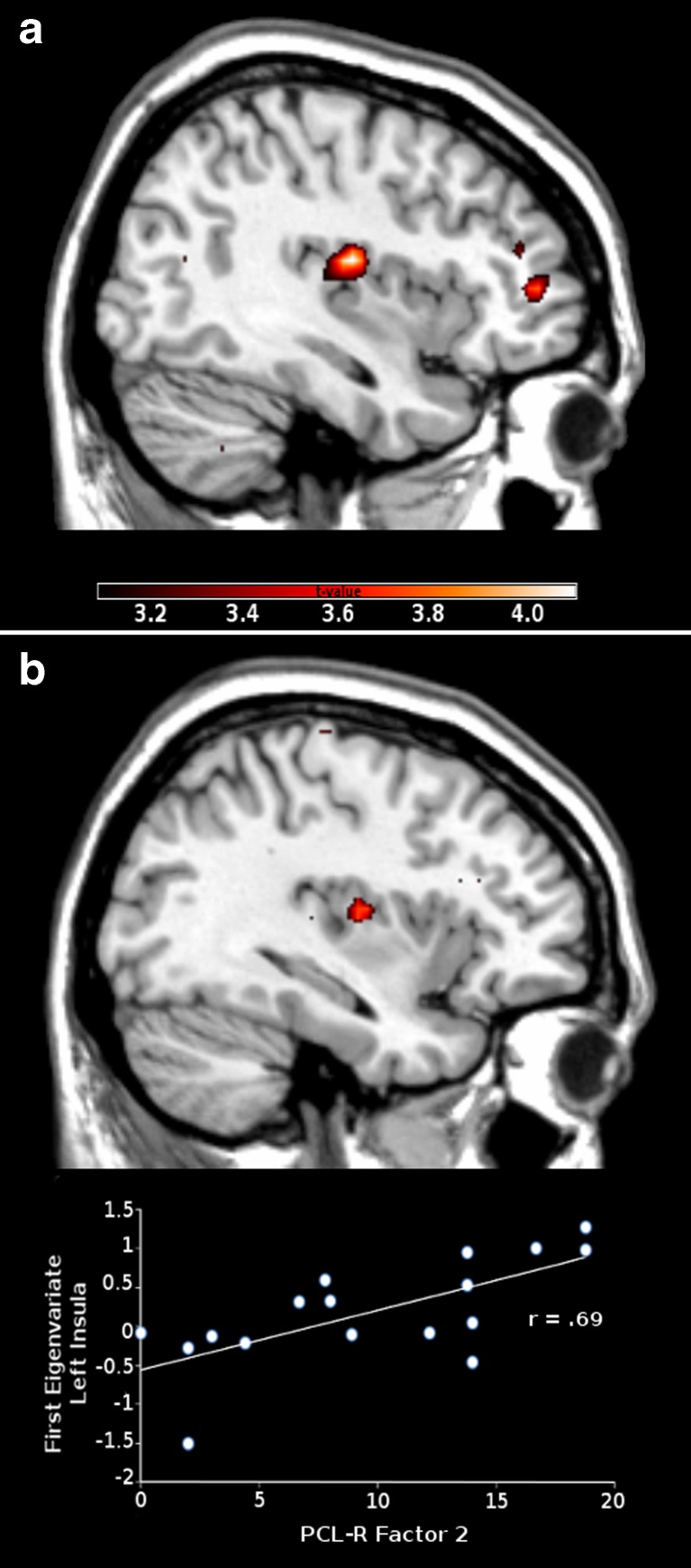
Increased insula activation in violent offenders relative to controls in response to approaching men (**a**), and positive correlation between PCL-R Factor 2 scores and insula activation (**b**). Footnote: PCL-R Factor 2 (Hare Psychopathy Checklist-Revised - chronically unstable, antisocial and socially deviant lifestyle)

Three interaction effects reached statistical significance (Group x Motion, Poser gender x Motion, and Group x Poser gender x Motion). The post-hoc t-contrasts showed that offenders relative to controls responded with greater left insula activation in the approach condition (interaction effect: Group x Motion), and with greater right insula activation when they were approached by men (interaction effect: Group x Poser gender x Motion; see Fig. 1). All participants were characterized by increased activation of the left amygdala and the inferior parietal region when they were approached by men (Poser gender x Motion).

The whole brain analysis revealed significant effects for Motion (see supplementary Table S2).

The regression analyses indicated a positive association between Factor 2 of the PCL-R and insular activation (MNI coordinates x,y,z: -36, −9, 12, *t* = 3.71, p_FWE_ = .049, cluster size =54) for the contrast Men > Women: Approaching > Static, but only in the offender group (see Fig. 1). The correlation between PCL-R Factor 1 and activation of the insula was not significant.

We had also conducted an exploratory voxel-based-morphometry analysis. The two groups did not differ in grey matter volume, neither when using the whole brain approach, nor in the ROIs.

## Discussion

We investigated neuronal and affective responses to PS intrusion in a group of high-risk violent offenders. Relative to non-criminal controls, the offenders showed greater insula activation to approaching persons, especially when the intruder was male. Well-known insular functions include interoceptive awareness as well as affective experience, especially arousal (Critchley et al. 2004). Valentini (2010) suggested that insular activation is a general neuronal correlate of potential threat and harm detection in PS. In order to demonstrate this function, Schaefer et al. (2012) presented their subjects with different videos that showed a tool coming closer to the participants’ hands (entering their peripersonal space), touching their fingers, and compared these two conditions with actual touch. All conditions involved the insula, and there was a significant overlap of activation for real touch and observed touch. Likewise, Lloyd et al. (2006) demonstrated increased insula activity in response to observing a painful versus non-painful stimulus without any tactile input. During fMRI recording, the participants watched while a visible rubber hand placed over their real hand was either touched with a sharp or a blunt probe. Thus, the insula was important for the motivational-affective processing of PS intrusion.

This interpretation is in line with the correlational findings of the present study. We observed a positive association between offenders’ scores on the PCL-R Factor 2 and insular activation during PS intrusion by men. In contrast, Factor 1 showed no association. Whereas the first facet of psychopathy describes a manipulative orientation, lack of empathy and emotional detachment, the second facet corresponds to an impulsive form of aggression (Hare 2003). In line with this conception, previous MRI investigations have shown that Factor 2 is associated with reactive anger, criminality, and impulsive violence (e.g., Leutgeb et al. 2015). Therefore, it appears very likely, that the offenders perceived the approach by men as a hostile act, especially when they had obtained high scores on Factor 2.

In agreement with previous investigations (Holt et al. 2014; Schienle et al. 2015) we were able to show that approaching faces generally activated a fronto-parietal network. The frontal area consisted of the premotor cortex, a key region for PS representation (e.g., Brozzoli et al. 2014). Neurons within this area (especially the ventral part) respond to objects moving toward the head and the body. They are direction-selective with stronger responses to approaching stimuli (Holt et al. 2014). In the present study, the zooming-in procedure gave the impression that the other face would come closer to one’s own face almost touching it. Previous neuroimaging studies showed that seeing someone being touched (e.g. viewing one’s own face being touched) is able to elicit activation of the premotor cortex, which can be considered part of a system that monitors stimuli in the PS including those that could collide with the body (Cardini et al. 2010).

Other approach-sensitive areas included the primary somatosensory cortex (SI) and the superior and inferior parietal region. The SI in the postcentral gyrus is the main sensory receptive area for the sense of touch. Several fMRI investigations demonstrated that not only actual but also observed touch and the observation of a stimulus which enters one’s own personal space is able to activate SI (e.g., Schaefer et al. 2012). Approach-biased activation in the superior parietal cortex has also been described before. This response was even content-specific and more pronounced to faces compared to approaching objects, such as cars (Holt et al. 2014).

The inferior parietal region is known to play a crucial role in the early integration of visual information with somatosensory, proprioceptive and vestibular signals. Previous investigations revealed that visual processing of noxious objects in personal space activated this area (Lloyd et al. 2006). The authors concluded that in many situations, it is survival-relevant for an organism to track potential threats in terms of their spatial proximity to the body by means of visuo-motor representations that are dynamically sensitive to events in PS.

We were not able to find that simulated proximity of another person generally provoked amygdala activation (Kennedy et al. 2009; Schienle et al. 2015). However these previous experiments had used different PS methods, only studied female participants, or presented affective facial expressions (angry, disgusted faces) and had not considered the gender of the approaching person. In the present study, amygdala recruitment was only seen when the approaching person was male. The amygdala is central for the control of defensive and aggressive responses to threat (LeDoux 2014). PS intrusion by another man might impose greater danger, especially when considering that the participants preferred greater personal distance to a man than to a woman as indexed by the drawn PS bubble.

We have to mention the following limitations of our study. We recruited only men because they represent the majority of violent offenders. Consequently, our findings cannot be generalized to women. In addition, the sample size was relatively small. However, we only studied non-psychopathic offenders without current comorbid mental disorders (with the exception of antisocial personality disorder) in order to be able to trace back the observed findings to the violent and aggressive behavior.

It also has to be noted that the chosen self-report measure of PS (drawn bubble) did not differentiate between offenders and controls. Therefore, a more ecologically valid PS assessment, such as the ‘Stop Distance Task’ might be helpful (Kaitz et al. 2004). Here, the experimenter slowly walks toward the subject, maintaining a neutral facial expression, and asks him/her to indicate ‘STOP’, when he/she starts to feel uncomfortable.

Future studies should extent the paradigm by including a withdrawal condition as well as different affective expressions. This will allow us to further deepen our knowledge about altered PS processing in violent offenders and the underlying neuronal correlates. Also, new intervention methods which aim at changing intrusion sensitivity might be tested and neuropsychologically validated.

In conclusion, this fMRI study is the first one to investigate the neuronal basis of PS intrusion in high-risk violent offenders. We were able to identify increased insular sensitivity to reductions of personal distance within this group, which possibly indexes their hostile attribution bias.

## Electronic supplementary material

Table S1 (DOCX 16 kb)

Table S2 (DOCX 26.5 kb)
