# Supplementary material for: Neuronal correlates of personal space intrusion in violent offenders
Source: Brain Imaging Behav. 2016 Mar 2;11(2):454–60. doi: 10.1007/s11682-016-9526-5 (PMC5408037; doi:10.1007/s11682-016-9526-5)
Supplement: Supplementary file 1 — (DOCX 16 kb) [file 11682_2016_9526_MOESM1_ESM.docx]

**Table S1: Affective ratings for the pictures**

| **Poser gender** | **Rating** | | **Offenders**  **M (SD)** | **Controls**  **M (SD)** |
| --- | --- | --- | --- | --- |
| **Static Faces** | |  | | |
| **Male** | Arousal | | 2.37 (1.46) | 2.47 (1.59) |
|  | Valence | | 3.87 (1.96) | 3.72 (1.86) |
|  |  | |  |  |
| **Female** | Arousal | | 2.15 (1.54) | 2.33 (1.36) |
|  | Valence | | 3.34 (1.28) | 3.52 (1.26) |
|  |  | |  |  |
| **Approaching Faces** | |  | | |
| **Male** | Arousal | | 3.03 (1.80) | 2.66 (1.54) |
|  | Valence | | 4.59 (2.07) | 3.91 (1.44) |
|  |  | |  |  |
| **Female** | Arousal | | 2.18 (1.15) | 2.61 (1.53) |
|  | Valence | | 3.12 (1.94) | 3.05 (1.46) |
