# Supplementary material for: Neuronal correlates of personal space intrusion in violent offenders
Source: Brain Imaging Behav. 2016 Mar 2;11(2):454–60. doi: 10.1007/s11682-016-9526-5 (PMC5408037; doi:10.1007/s11682-016-9526-5)
Supplement: Supplementary file 2 — (DOCX 26.5 kb) [file 11682_2016_9526_MOESM2_ESM.docx]

**Table S2: Results of the analysis of variance (whole brain approach)**

|  | | **H** | **x** | **y** | **z** | **F** | **Post-hoc**  **t-tests** | | | | **p(FWE)** |  |
| --- | --- | --- | --- | --- | --- | --- | --- | --- | --- | --- | --- | --- |
| **Main Effect MOTION** | |  |  |  |  |  |  | |  | | | |
| **Approaching > Static** |  | | | | | | | | | | | |
| Lingual gyrus | | L | -5 | -80 | -2 | 211.31 |  | 14.54 | | <0.001 | | |
| Calcarine fissure | | R | 5 | -77 | 5 | 181.22 |  | 13.46 | | <0.001 | | |
| Cuneus | | R | 12 | -90 | 18 | 139.28 |  | 11.80 | | <0.001 | | |
| Superior occipital gyrus | | L | -18 | -84 | 21 | 114.55 |  | 10.70 | | <0.001 | | |
| Middle occipital gyrus | | L | -42 | -72 | 9 | 102.98 |  | 10.15 | | <0.001 | | |
| Middle temporal gyrus | | R | 50 | -62 | 5 | 79.01 |  | 8.88 | | <0.001 | | |
| Inferior occipital gyrus | | R | 41 | -68 | -8 | 65.83 |  | 8.11 | | <0.001 | | |
| Middle cingulate gyrus | | L | -12 | -21 | 42 | 61.12 |  | 7.82 | | <0.001 | | |
| Precentral gyrus | | L | -33 | -3 | 48 | 53.57 |  | 7.32 | | 0.001 | | |
| Fusiform gyrus | | L | -33 | -51 | -18 | 45.55 |  | 6.75 | | 0.002 | | |
| Superior parietal lobule | | L | -30 | -51 | 51 | 45.43 |  | 6.74 | | 0.002 | | |
| Precuneus | | R | 11 | -44 | 50 | 44.52 |  | 6.67 | | 0.003 | | |

**H: hemisphere, x,y,z: MNI coordinates, F-values of analyses of variance, post-hoc t-tests with p (corrected for family-wise error (FWE)).**
